# Knowledge, attitudes and behaviour of Egyptians towards antibiotic use in the community: can we do better?

**DOI:** 10.1186/s13756-023-01249-5

**Published:** 2023-05-24

**Authors:** Lina Maarouf, Mohamed Amin, Benjamin A. Evans, Alaa Abouelfetouh

**Affiliations:** 1 Department of Microbiology and Immunology, Faculty of Pharmacy, Alexandria University, 1 Khartoum Sq, Azarita, Alexandria 21521 Egypt; 2 Norwich Medical School, University of East Anglia, Norwich, UK; 3 Department of Pharmacy Practice and Clinical Pharmacy, Faculty of Pharmacy, Alamein International University, New Alamein City, 51718 Egypt; 4 Department of Microbiology and Immunology, Faculty of Pharmacy, Alamein International University, New Alamein City, 51718 Egypt

**Keywords:** Antibiotic resistance, Awareness campaign, Educational intervention, Common cold, The theory of planned behaviour

## Abstract

**Background:**

Infectious diseases are among the leading causes of death worldwide. This is concerning because of the increasing capacity of the pathogens to develop antibiotic resistance. Antibiotic overuse and misuse remain the main drivers of resistance development. In the USA and Europe, annual campaigns raise awareness of antibiotic misuse hazards and promote their judicial use. Similar efforts are lacking in Egypt. This study assessed the knowledge of the public in Alexandria, Egypt of antibiotic misuse risks and their habits towards antibiotic use, in addition to conducting a campaign to increase awareness of the safe use of antibiotics.

**Methods:**

A questionnaire assessing knowledge, attitudes and behaviour towards antibiotics was used to collect responses from study participants at various sports clubs in Alexandria in 2019. An awareness campaign to correct misconceptions and a post awareness survey followed.

**Results:**

Most of the participants were well-educated (85%), in their middle age (51%) and took antibiotics last year (80%). 22% would take an antibiotic for common cold. This dropped to 7% following the awareness. There was a 1.6 time increase in participants who would start an antibiotic on a healthcare professional’s advice following the campaign. A 1.3 time increase in participants who would finish an antibiotic regimen was also observed. The campaign made all participants recognize that unwise antibiotic use is harmful to them or others; and 1.5 more participants would spread the word about antibiotic resistance. Despite learning of the risks of antibiotic use, there was no change in how often participants thought they should take antibiotics.

**Conclusions:**

Although awareness of antibiotic resistance is rising, some wrong perceptions hold fast. This highlights the need for patient and healthcare-tailored awareness sessions as part of a structured and national public health program directed to the Egyptian population.

**Supplementary Information:**

The online version contains supplementary material available at 10.1186/s13756-023-01249-5.

## Background

Antimicrobial resistance (AMR) represents a serious problem described by the World Health Organization (WHO) as “a global public health concern” [1]. AMR has dire consequences worldwide as it increases morbidity and mortality rates from bacterial infection and mandates quick interventions to mitigate the problem [2]. In the last three decades, the rate of approval of new antibiotics has slowed while antibiotic resistant bacterial pathogens have continued to emerge [3]. Moreover, pathogens keep acquiring more resistance determinants leading to the emergence of multidrug-resistant (MDR) and even extensive drug-resistant (XDR) and pan drug-resistant (PDR) bacteria which are resistant to all antibiotics [4].

The emergence of these resistant bacteria leads to a socioeconomic burden worldwide due to increased healthcare costs as a result of using more expensive antimicrobials and prolonged hospital stays, in addition to lost productivity. It is estimated that the annual health burden of the treatment of MDR bacterial infections in the USA is around $20 billion, with $35 billion lost due to reduced productivity [3], and a global annual death toll of 700,000 in 2014 that is expected to increase to 10 million by 2050 [5].

Research from around the globe indicates high rates of injudicious use of antibiotics by the public. Practices related to injudicious use in the literature include the use of left over antibiotics [6, 7] and antibiotic non-adherence usually due to remission of symptoms [8]. Further, the pressure made by patients who request antibiotics from clinicians has been shown to be one of the factors driving unwarranted provision of antibiotics by clinicians such as physicians [9] and pharmacists [10]. Antibiotic abuse and misuse in the form of overuse or underuse remain some of the most important factors contributing to resistance development particularly in developing countries (reviewed in [11]). A study investigating the drivers of antibiotic use and misuse in the community found patient behaviour to be an important driving factor. This takes the form of patients putting pressure on a physician to prescribe an unnecessary antibiotic either directly by demanding an antibiotic or indirectly when questioning the absence of an antibiotic in a prescription [12, 13]. Moreover, many patients believe they have the right to self-prescribe an antibiotic or get one from friends and family [14]. About 50% of antibiotic use is done improperly in the form of using the wrong agent or the wrong dose or duration (reviewed in [11]).

In Egypt, antibiotic misuse and overuse led to a high prevalence of MDR bacteria among the population (reviewed in [15]). Most studies comment on the resistance rates among a particular MDR pathogen rather than on the general prevalence of MDR organisms. El Kholy et al. found that around 70% of *Staphylococcus aureus* isolates were resistant to a wide range of antibiotic classes. Another study reported fluoroquinolone resistance among methicillin-resistant *S. aureus* (MRSA) isolates from Alexandria, Egypt to range between 78% and 96% [16]. Among Gram negative pathogens, 94% of *E. coli* isolates were resistant to ampicillin [17] and upward of 70% of *Acinetobacter baumannii* isolates from Alexandria Main University Hospital were MDR [18]. However, only a small number of studies reported the percentage of antibiotic misuse among Egyptian community pharmacies. Abdelaziz et al. reported that 98% of the visited pharmacies dispensed an antibiotic as an over the counter (OTC) drug to treat ‘flu symptoms’ [19]. In another report, Dooling et al. mentioned that 64% and 81% of participating physicians and pharmacists, respectively, from Minya, Egypt were prescribing antibiotics for patients with symptoms consistent with a common cold [20]. Moreover, a “cold group” that is a combination of pills, that may contain antibiotics, and that is commonly sold in some Egyptian pharmacies to treat common cold was provided to simulated clients in 28.7% of the cases. In all of these, the cold group contained one or more antibiotic pills [21].

Patient pressure has been identified as an important driver for injudicious prescribing and dispensing of antibiotics, yet a lone campaign in Minya, Egypt promoted the appropriate use of antibiotics to treat respiratory tract infections among prescribers and the general public [22]. The aim of the current work was to assess the knowledge of antibiotic resistance among the public in Alexandria, Egypt and their attitudes towards antibiotic use. Moreover, an awareness campaign was designed to educate the participants on appropriate antibiotic use principles. The knowledge was re-assessed at the conclusion of the campaign to measure its effectiveness. In order to achieve these aims we targeted the parents of children attending sports clubs, as this would allow us to repeatedly interact with the same individuals over the course of several weeks. To the best of the authors’ knowledge, this is the second published awareness campaign in Egypt and the first in Alexandria to address wise antibiotic use among the general public.

## Methods

A questionnaire was used to collect responses from study participants who were the parents of children playing sports at different sports clubs in Alexandria, Egypt. The study was conducted over three months between July and September 2019. It was divided into three phases. Phase one consisted of a general survey about participant’s antibiotic use and knowledge of antibiotic resistance. Phase two consisted of an antibiotic awareness campaign to guide people against the misuse of antibiotics and its consequences on antibiotic resistance, and phase three consisted of a post awareness questionnaire to assess the effectiveness of the awareness sessions. The awareness sessions were delivered by Alexandria University clinical pharmacy students and took the form of informal chats with the participants. As part of the sessions, the participants were given some flyers (Additional File 1) stressing the awareness messages. The students took turns delivering the messages and used the flyers as guiding points.

Ethical approval for the study was obtained from the Faculty of Medicine and Health Sciences Research Ethics Committee, University of East Anglia (Reference 201,819–102), and The Research Ethics Committee at the Faculty of Pharmacy, Alexandria University prior to study commencement.

### Sampling

Convenience sampling was used to recruit the participants. No specific sample size was designated before study commencement. The sample size was determined in light of the number of students contributing to the campaign. The students set up working stations on weekdays and weekends in each of the sports clubs they visited and approached all adults who came in contact with them. All potential study participants were provided with a participant information sheet prior to taking part in the study, and informed consent was obtained from all participants for the collection and use of their data before beginning the study. The participants were then given the paper-based questionnaire by the students. The participants returned the completed questionnaire back to the students when they were done.

### Design of the questionnaire

The questionnaire (Additional File 2 shows the Arabic version and Additional File 3 shows the English translation) covered the demographic and professional data of the participants and then was divided into two parts. The first part consisted of six questions collecting data about past antibiotic use. The second part contained 13 questions about knowledge and attitudes towards antibiotics and antibiotic resistance. The participants were asked to fill both parts of the questionnaire at phase one, and just the second part at phase three following the awareness campaign. The full questionnaire needed an average of five minutes to be completed. A pilot study covering over 500 participants had been carried out prior to current data collection.

The questionnaire was anonymous, the demographic data collected can’t be used to determine the identity of the participants, and therefore the data from phase one and phase three can’t be linked to individuals.

### Data analysis

The data were tabulated and statistically analysed using Fisher’s exact test and Chi squared test, where the results were considered significant at a *p* value of ≤ 0.05. The Independent variable was the education, and the dependent variables were knowledge and attitudes towards antibiotic use and resistance. Categorical variable was classified to Zero (unexposed to education), 1 (exposed to education). Data showing significant differences were subsequently analysed by binary or multinomial logistic regression in SPSS (v. 28.0.1.1) to obtain odds ratios. For multinomial regression, the reference category was set as the largest category for each dataset.

## Results

A total of 626 sports club attendees were approached and they all agreed to participate in the study making the response rate 100%. The majority of the participants (51%) were aged 31 to 50 years old, 76% of the participants were females and 85% were university graduates holding a bachelor or a higher degree.

Surveying the history of antibiotic use showed that more than 80% of the participants took antibiotics last year at least once, with 46% taking antibiotics 2 to 3 times. Of those who had taken antibiotics, 80% took them on the advice of a healthcare professional (62% from a doctor, 18% from a pharmacist), while 20% took them after advice from non-healthcare professional sources (13% decided themselves, 6% from a family member, 1% from a friend) (Table 1). On the other hand, more than 50% of the participants said that they were informed by their physician that they don’t need an antibiotic to treat their symptoms, yet > 80% of the participants took antibiotics in the previous year.

**Table 1:** Participant demographics and antibiotic use in the previous year

	Number	Percentage
**Age group (years)** 18–30 31–50 51–70 > 70	192 320 92 22	30.7 51.1 14.7 3.5
**Gender** Female Male Prefer not to say	478 146 2	76.4 23.3 0.3
**Level of education** Postgraduate Bachelor High school graduate Middle school graduate None	95 435 85 10 1	15.2 69.5 13.6 1.6 0.2
**Antibiotic use in last year** Once 2–3 times ≥ 4 times	184 241 94	35.5 46.4 18.1
**Who advised you to take the antibiotics?** Doctor Pharmacist Nurse Friend Family member Self Other	345 99 0 6 31 70 2	62.4 17.9 0 1.1 5.6 12.7 0.4

Looking at participant knowledge of antibiotic use, 35% of the participants believed that more expensive antibiotics would be more effective. This percentage was almost halved following the campaign (binary logistic regression, OR = 0.431, 95% CI = 0.196–0.945, *p* = 0.036). A total of 78% of the participants thought that they should not take antibiotics for a common cold; this percentage increased to 93% after the campaign (binary logistic regression, OR = 0.255, 95% CI = 0.078–0.837, *p* = 0.024). Yet, almost half of the participants would start an antibiotic based on their own assessment of their symptoms versus 34% who would start an antibiotic following advice from a physician or pharmacist. These percentages changed to 20% and 55% following the awareness campaign (Chi squared test, *p* < 0.001). Nevertheless, 64% of the participants knew that they should complete the antibiotic regimen, which increased to 86% as a consequence of the awareness campaign (Chi squared test, *p* < 0.001) (Table 2).

**Table 2:** Participant knowledge and attitudes towards antibiotics before and after the awareness sessions

Participant knowledge	Before awareness		After awareness		*p* value	Regression			
	n	%	n	%		*p* value^a^	Odds ratio	Lower 95% CI	Upper 95% CI
**Do you believe more expensive antibiotics are more likely to make you feel better?** Yes No	216 407	34.7 65.3	8 35	18.6 81.4	0.011455	0.036	0.431	0.196	0.945
**Do you believe you have to take an antibiotic whenever you have an infection, like a cold?** Yes No	139 483	22.3 77.7	3 40	7 93	0.004288	0.024	0.255	0.078	0.837
**When you get a cold, when do you think you should start taking antibiotics?** I don’t take them When feeling the first symptoms of a cold After cough and sputum appear After the colour of mucous changes After a doctor or pharmacist tells me to take one I have never had a cold I don’t know Others	88 71 124 105 207 1 18 10	14.1 11.4 19.9 16.8 33.2 0.2 2.9 1.6	11 3 4 2 24 0 0 0	25 6.8 9.1 4.5 54.5 0 0 0	2.67021E-07	0.845 0.108 0.02 0.015 Ref - - -	1.078 0.364 0.278 0.164 - - - -	0.506 0.107 0.094 0.038 - - - -	2.296 1.247 0.821 0.708 - - - -
**When you have been taking antibiotics, when do you think you should stop the antibiotic treatment?** When I feel better After I finish the full course I have never taken antibiotics I don’t know	202 397 18 5	32.5 63.8 2.9 0.8	5 37 1 0	11.6 86 2.3 0	6.41626E-05	0.006 Ref 0.619 -	0.266 - 0.596 -	0.103 - 0.077 -	0.686 - 4.592 -
**Participant attitudes**	**Before awareness**		**After awareness**		***p*** **value**	** Regression**			
	**n**	**%**	**n**	**%**		**p value** ^**a**^	** Odds ratio**	** Lower 95% CI**	**Upper 95% CI **
**Would you ask a doctor to prescribe an antibiotic if you believe you need one, even if the doctor did not think it was needed?** Yes No	109 513	17.5 82.5	3 40	7 93	0.030886	0.087	0.353	0.107	1.162
**Would you buy antibiotics to use without being told you need them by a doctor?** Yes No	208 413	33.5 66.5	7 34	17.1 82.9	0.013828	0.035	0.409	0.178	0.938
**Do you think you should be able to buy antibiotics whenever you want them?** Yes No	188 433	30.3 69.7	11 30	26.8 73.2	0.642698	nd	nd	nd	nd

^a^Ref indicates this is the reference category in multinomial logistic regression analyses. nd, not done as initial analysis reported no significant difference. Dashes (-) in *p* value, OR and 95% CI columns for multinomial regression represent non-returned values due to insufficient data. Answers to some questions were missing

Concerning participants’ attitudes towards antibiotic use, only 18% reported that they would put pressure on the physician to prescribe an antibiotic, which dropped to 7% after the campaign (Fisher’s exact test, *p* = 0.031; binary logistic regression, OR = 0.353, 95% CI = 0.107–1.162, *p* = 0.087). While there was also a significant drop in the number of participants who would buy antibiotics to use even if a doctor hadn’t told them they needed them (34% before the awareness campaign vs. 17% after; binary logistic regression, OR = 0.409, 95% CI = 0.178–0.938, *p* = 0.035), there remained a substantial number of people who would still purchase antibiotics. This was further reflected in the fact that there was no significant difference in the number of participants who felt they should be able to buy antibiotics whenever they want them, with almost one in three participants wanting the freedom to do so (Table 2).

Regarding antibiotic resistance, 78% of participants had already heard about the problem even before the campaign; this occurred through interaction with a healthcare practitioner in 47% of cases and through the media in 33% (Table 3). It is of note that as many people had heard about antibiotic resistance from a physician or a nurse as had heard about it from the media (33% in both cases). Despite hearing about antibiotic resistance, about 11% thought that unwise antibiotic use won’t have a harmful effect. Following the awareness campaign, 100% of the participants appreciated that resistance is a problem for everyone now and in the future (Chi squared test, *p* < 0.001). As for participant’ attitudes towards antibiotic resistance, 87% have now told others about antibiotic resistance versus just 59% before the campaign (binary logistic regression, OR = 4.729, 95% CI = 1.818–12.301, *p* = 0. 001), yet the campaign does not seem to have changed how often people would take an antibiotic (Table 3).

**Table 3:** Participant knowledge and attitudes towards antibiotic resistance before and after the awareness sessions

Participant knowledge	Before awareness		After awareness		*p* value	Regression			
	n	%	n	%		*p* value^a^	Odds ratio	Lower 95% CI	Upper 95% CI
**How many times have you heard about antibiotic resistance?** Many times A few times Only once I don’t remember hearing about antibiotic resistance	388 43 50 136	62.9 7 8.1 22	30 0 11 2	69.8 0 25.6 4.7	8.97963E-13	Ref - 0.006 0.024	- - 2.845 0.19	- - 1.343 0.045	- - 6.03 0.806
**Where have you heard about antibiotic resistance?** From a physician or nurse From a pharmacist From friends or family At school/college/university In the media (television, radio, newspapers, magazines etc.) Others	167 69 70 29 166 2	33.2 13.7 13.9 5.8 33 0.4	6 19 5 0 8 2	15 47.5 12.5 0 20 5	3.41269E-32	0.594 0.00009 0.503 - Ref 0.004	0.746 5.714 1.482 - - 20.75	0.253 2.388 0.468 - - 2.581	2.195 13.672 4.689 - - 166.828
**How widespread do you think antibiotic resistance is?** Mostly found in rich countries Mostly found in poor countries Worldwide	52 153 269	11 32.3 56.8	4 9 26	10.3 23.1 66.7	0.110692	nd	nd	nd	nd
**What do you think will happen if you use antibiotics unnecessarily?** Nothing Antibiotic-resistant infections will personally affect me Antibiotic-resistant infections will affect others in the future Antibiotic-resistant infections will affect me, and others in the future	54 211 24 203	11 42.9 4.9 41.3	0 18 0 21	0 46.2 0 53.8	0.000175	- Ref - 0.566	- - - 1.213	- - - 0.628	- - - 2.342
**Participant attitudes**	**Before awareness**		**After awareness**		***p*** **value**	** Regression**			
	**n**	**%**	**n**	**%**		**p value** ^**a**^	**Odds ratio **	** Lower 95% CI**	**Upper 95% CI **
**Has hearing about antibiotic resistance changed how often you think you should take antibiotics?** Yes – I think I should take them more Yes – I think I should take them less No	36 352 87	7.6 74.1 18.3	3 29 5	8.1 78.4 13.5	0.46244164	nd	nd	nd	nd
**Have you told anyone else about antibiotic resistance?** Yes No	289 201	59 41	34 5	87.2 12.8	7.4E-06	0.001	4.729	1.818	12.301

^a^Ref indicates this is the reference category in multinomial logistic regression analyses. nd, not done as initial analysis reported no significant difference. Dashes (-) in *p* value, OR and 95% CI columns for multinomial regression represent non-returned values due to insufficient data. Answers to some questions were missing

## Discussion

Antibiotic resistance, which is a worldwide crisis, is a crucial problem; and urgent action is needed to resolve it. One of the important pillars in combatting AMR is improving public knowledge of antibiotic use [23]. Globally, many antibiotic awareness campaigns have been conducted to increase public understanding of the risks around the misuse of antibiotics and the threat from antibiotic resistance. For instance, European Antibiotic Awareness Day (EAAD), which was launched in 2008 is an annual event co-ordinating public engagement activities across Europe [24], and World Antibiotic Awareness Week, which was firstly introduced by WHO in 2015, is held in the middle of November annually [25]. Therefore, this study aimed to assess public knowledge and then determine the impact of an awareness campaign designed to provide the public with information about appropriate antibiotic use and the problem of resistance. Promising findings from this study are in line with recent research in Egypt showing an improvement in prescribing habits, attitude and belief scores for physicians, pharmacists, and patients regarding antibiotic use following a campaign in Minya, a governorate in Southern Egypt [22].

Although the sample size was not pre-determined and it mainly depended on the number of students delivering the survey and awareness campaign, given the large number of participants in the study and the significance of findings we do not anticipate that inadequate power was to be an issue of concern. In general, most of the tested population (85%) used antibiotics throughout the last year, which is almost the same percentage (89%) described in a recent WHO report [26] that studied antibiotic use in different countries including Egypt. 80% of the participants had been prescribed antibiotics by healthcare professionals, and only 13% were self-medicated; this percentage is lower than previously reported by WHO (26%) [26] and in an Egyptian study on non-medical students (39%) [27]. This could be explained by the high percentage of well-educated participants in our study who also tend to be wealthier. As such, they could afford to consult with healthcare professionals rather than being forced to self-medicate.

Moreover, one third of participants believed that the more expensive an antibiotic is, the more effective it is. This could be explained by the perception in general that any product with a higher price will be higher in quality, neglecting its actual properties [28]. However, as a result of the campaign, this percentage was almost halved.

In total 22% of the participants believed that antibiotics could be used to treat the common cold, which is much lower than found in the WHO’s report (76%) [26] and close to what was published in a survey in 2017 in England (15%) [29]. In these studies, the participants may not have been aware that the common cold is a viral infection that could not be treated by antibiotics. Our awareness campaign was particularly effective at correcting this misconception, with only 7% of people saying they would take an antibiotic for a cold after taking part. Similarly, concerning the completion of the antibiotic course, 64% of participants believed that they should complete their antibiotic course, which is high compared to the WHO report (41%) [26]. However, this value increased to 86% after the campaign, which comes near the rate described in the English report (87%) [29]. The high level of awareness of the need to complete the antibiotic course seen in the current study might be explained by the high percentage of well-educated participants.

The final part of the survey assessed the participants’ knowledge about antibiotic resistance. We found that almost 70% of the participants had already heard about antibiotic resistance more than once and from different sources, mainly healthcare professionals and from media sources. This figure is almost 3 times higher than in the WHO report (22%) [26] and around twice as high as previously found in Egypt (40%) [27]. This shows that the repetition of the message alone might not be enough to promote behaviour change. Moreover, whereas 11% of participants thought that there will be no harmful effects from antibiotic resistance in the future, this concept has totally disappeared after the awareness campaign, in a demonstration of the health belief model [30]. Overall, these data show that even in a population that is relatively well informed, awareness campaigns can help change public understanding of the problems posed by AMR. This is echoed by a systematic review showing that interventions to raise antibiotic awareness among the public in the USA improved knowledge, attitudes and behaviour [31].

While the awareness campaign was quite effective at changing the views and understanding of participants (views were changed in 10 out of 13 questions) there is still clearly work to be done to improve public knowledge. For example, despite the awareness campaign, 1 in 5 participants still believed a more expensive antibiotic would be better for them, and would self-medicate, while 8% believed they should be taking more antibiotics rather than fewer. It is possible that these represent people who are more resistant to change. Our study population consisted largely of adults of an age at which their views and behaviours will have been embedded for some time, and which are harder to change than those of much younger people [32]. When properly designed, education on antibiotic resistance may be more effective in children [33], who are more open to new concepts and have had less exposure to pre-existing misconceptions. This work also stresses the importance of a multi-tiered approach combining educational interventions and peer influence targeting healthcare professionals and patients, as well as regulatory enforcement. It is of particular note that of the three questions for which there was no difference in responses following the awareness campaign, two were related to direct effects on individual choice and freedom – there was no change in the number of people who felt they should be able to buy antibiotics whenever they liked, and no change in how often people felt they personally should take antibiotics. This would appear to be in contradiction to the answers given to other questions, such as 100% of participants post awareness believing that unnecessary antibiotic use will adversely affect them and/or others. It is likely that this is due to the phenomenon that while people can understand an issue and agree that certain behaviours at a population level need to change, they don’t believe that they should be restricted from access to a certain service if it is deemed inappropriate by public health standards. This responds to the perceived behavioural control construct of the theory of planned behaviour (TPB) explained below [34]. When designing awareness campaigns, consideration should be given to how people can be engaged at an individual, more personal level to enable them to identify how they could alter their own behaviour. A focus of future campaigns may be on potential harm of nonindicated antibiotics to the targeted individual rather than societal harm resulting from antibiotic resistance in general [35].

The findings of the current study are aligned with the TPB that explains all behaviours that people control and that has been used to explain many health behaviours. It consists of a number of key constructs: (a) attitudes, (b) subjective norms and (c) perceived behavioural control. These components determine the behavioural intention of a person [36]. In the current context, attitudes are exemplified by using or asking for antibiotics; the awareness campaign resulted in reduction in the number of participants that would take an antibiotic whenever they have an infection which is indicative of improved attitudes. Subjective norms are explained here by the participants’ perception of whether people important to them would use or ask for an antibiotic. In the current study the finding that the participants were more likely to tell others about antibiotics following the educational intervention is an indication of a positive impact on their subjective norms.

Finally, perceived behavioural control is exemplified here by the difficulty level of obtaining an antibiotic. The finding that following the awareness session participants were not more likely to embrace a restriction to their ability to obtain antibiotics on demand responds to the perceived behavioural control. In future campaigns this can be addressed by stressing the benefits of restricting antibiotic use in limiting antibiotic resistance development.

## Conclusions

In conclusion, the tested population had some good pre-existing knowledge and attitudes to antibiotics and antibiotic resistance, but this was still increased as a result of the awareness campaign, with some incorrect perceptions corrected. However, some misconceptions persisted in a small proportion of the population, and this proportion would likely be higher in a population with less pre-existing knowledge. Hence, we recommended increasing awareness campaigns for the public with customized engagement materials and activities that are more likely to enable individuals to identify changes they could make in their own behaviour.

To the best of the authors’ knowledge this is the second published campaign to address the wise use of antibiotics among the general public in Egypt and the first in Alexandria.

The main limitation of this study is that convenience sampling was used to enroll participants resulting in a relatively high level of education. Future studies might address this by replicating and expanding on this work with patients of lower educational levels. Selection bias resulting from potential differences between the groups before and after the awareness campaign is a potential limitation. Second, social desirability bias might have impacted the reported rates. Third, the study used a single approach to improve antibiotic use and that is raising public awareness and didn’t address other forms and/or levels of interventions. Finally, despite targeting a specific group so that we could reassess their knowledge at subsequent visits to the clubs, it was difficult to persuade participants to re-do the survey leading to a much smaller group number following the awareness campaign. We do not anticipate differences among the before and after awareness groups in relation to key study variables. In future work, we need to consider how to better incentivise this.

## Electronic supplementary material

Below is the link to the electronic supplementary material.

Additional File 1. Examples of educational flyers used by the clinical pharmacy students in the awareness campaign.

Additional File 2. The Arabic version of the questionnaire used to assess the participants’ knowledge, attitudes and behaviour towards antibiotic use and antibiotic resistance.

Additional File 3. The English translation of the questionnaire used to assess the participants’ knowledge, attitudes and behaviour towards antibiotic use and antibiotic resistance.

## Data Availability

The datasets generated and analysed in the current study are available from the corresponding author upon reasonable request.

## List of Abbreviations

AMR: Antimicrobial Resistance
EAAD: European Antibiotic Awareness Day
XDR: Extensive Drug-Resistant
MDR: Multi Drug-Resistant
MRSA: Methicillin Resistant *Staphylococcus aureus*
OTC: Over The Counter
PDR: Pan Drug-Resistant
TPB: Theory of Planned Behaviour
UK: United Kingdom
USA: United States of America
WHO: World Health Organization

## References

[1] Organization WH. Antimicrobial Resistance: Global Report on Surveillance 2014 [Available from: https://apps.who.int/iris/handle/10665/112642.

[2] Aslam B, Wang W, Arshad MI, Khurshid M, Muzammil S, Rasool MH (2018). Antibiotic resistance: a rundown of a global crisis. Infect Drug Resist.

[3] Ventola CL (2015). The antibiotic resistance crisis: part 1: causes and threats. P T.

[4] Magiorakos AP, Srinivasan A, Carey RB, Carmeli Y, Falagas ME, Giske CG (2012). Multidrug-resistant, extensively drug-resistant and pandrug-resistant bacteria: an international expert proposal for interim standard definitions for acquired resistance. Clin Microbiol infection: official publication Eur Soc Clin Microbiol Infect Dis.

[5] Review on Antimicrobial Resistance. Tackling drug-resistant infections globally: final report and recommendations. 2016.

[6] Machongo RB, Mipando ALN (2022). I don’t hesitate to use the left-over antibiotics for my child” practices and experiences with antibiotic use among caregivers of paediatric patients at Zomba central hospital in Malawi. BMC Pediatr.

[7] Voidăzan S, Moldovan G, Voidăzan L, Zazgyva A, Moldovan H (2019). Knowledge, Attitudes and Practices regarding the use of antibiotics. Study on the General Population of Mureş County, Romania. Infect Drug Resist.

[8] Endashaw Hareru H, Sisay D, Kassaw C, Kassa R (2022). Antibiotics non-adherence and its associated factors among households in southern Ethiopia. SAGE Open Medicine.

[9] Sirota M, Round T, Samaranayaka S, Kostopoulou O (2017). Expectations for antibiotics increase their prescribing: causal evidence about localized impact. Health psychology: official journal of the Division of Health Psychology American Psychological Association.

[10] Amin MEK, Amine A, Newegy MS (2017). Perspectives of pharmacy staff on dispensing subtherapeutic doses of antibiotics: a theory informed qualitative study. Int J Clin Pharm.

[11] Aboulmagd E, Kassem MA, Abouelfetouh A, Ghazi I, Cawley M (2020). Global Landscape in Microbial Resistance. 21st Century Challenges in Antimicrobial Therapy and Stewardship. Frontiers in anti-infective agents.

[12] Sanchez GV, Roberts RM, Albert AP, Johnson DD, Hicks LA (2014). Effects of knowledge, attitudes, and practices of primary care providers on antibiotic selection, United States. Emerg Infect Dis.

[13] Stivers T, Mangione-Smith R, Elliott MN, McDonald L, Heritage J (2003). Why do physicians think parents expect antibiotics? What parents report vs what physicians believe. J Fam Pract.

[14] Byrne MK, Miellet S, McGlinn A, Fish J, Meedya S, Reynolds N (2019). The drivers of antibiotic use and misuse: the development and investigation of a theory driven community measure. BMC Public Health.

[15] Hashem RA, Yassin AS, Zedan HH, Amin MA (2013). Fluoroquinolone resistant mechanisms in methicillin-resistant *Staphylococcus aureus* clinical isolates in Cairo, Egypt. J Infect Dev Ctries.

[16] Alseqely M, Newton-Foot M, Khalil A, El-Nakeeb M, Whitelaw A, Abouelfetouh A (2021). Association between fluoroquinolone resistance and MRSA genotype in Alexandria, Egypt. Sci Rep.

[17] El Kholy A, Baseem H, Hall GS, Procop GW, Longworth DL (2003). Antimicrobial resistance in Cairo, Egypt 1999–2000: a survey of five hospitals. J Antimicrob Chemother.

[18] Abouelfetouh A, Torky AS, Aboulmagd E (2020). Role of plasmid carrying bla NDM in mediating antibiotic resistance among Acinetobacter baumannii clinical isolates from Egypt. 3 Biotech.

[19] Abdelaziz AI, Tawfik AG, Rabie KA, Omran M, Hussein M, Abou-Ali A et al. Quality of Community Pharmacy Practice in Antibiotic Self-Medication Encounters: A Simulated Patient Study in Upper Egypt. 2019;8(2). 10.3390/antibiotics8020035 PMC6627069 30939797

[20] Dooling KL, Kandeel A, Hicks LA, El-Shoubary W, Fawzi K, Kandeel Y (2014). Understanding antibiotic use in Minya District, Egypt: Physician and Pharmacist Prescribing and the factors influencing their Practices. Antibiot (Basel Switzerland).

[21] Amin ME, Amine A, Newegy MS. Injudicious Provision of Subtherapeutic Doses of Antibiotics in Community Pharmacies. Innovations in Pharmacy. 2017;8(1).

[22] Kandeel A, Palms DL, Afifi S, Kandeel Y, Etman A, Hicks LA (2019). An educational intervention to promote appropriate antibiotic use for acute respiratory infections in a district in Egypt- pilot study. BMC Public Health.

[23] Cross EL, Tolfree R, Kipping R (2017). Systematic review of public-targeted communication interventions to improve antibiotic use. J Antimicrob Chemother.

[24] Mason T, Trochez C, Thomas R, Babar M, Hesso I, Kayyali R (2018). Knowledge and awareness of the general public and perception of pharmacists about antibiotic resistance. BMC Public Health.

[25] Yasmeen BN (2016). The first World Antibiotic Awareness Week on Antibiotic Resistance. North Int Med Coll J.

[26] Organization WH. Antibiotic resistance: Multi-country public awareness survey. 2015.

[27] Mostafa A, Abdelzaher A. Is health literacy associated with antibiotic use, knowledge and awareness of antimicrobial resistance among non-medical university students in Egypt? A cross-sectional study. 2021;11(3):e046453. 10.1136/bmjopen-2020-046453 PMC8098941 33649060

[28] Shirai M (2015). Impact of “High quality, low Price” appeal on consumer evaluations. J Promotion Manage.

[29] McNulty CAM, Collin SM, Cooper E, Lecky DM, Butler CC (2019). Public understanding and use of antibiotics in England: findings from a household survey in 2017. BMJ Open.

[30] Rosenstock IM (1974). The Health Belief Model and Preventive Health Behavior. Health Educ Monogr.

[31] Burstein VR, Trajano RP, Kravitz RL, Bell RA, Vora D, May LS (2019). Communication interventions to promote the public’s awareness of antibiotics: a systematic review. BMC Public Health.

[32] Tucker JS, Klein DJ, Elliott MN (2004). Social Control of Health Behaviors: a comparison of Young, Middle-Aged, and older adults. The Journals of Gerontology: Series B.

[33] Appiah B, Anum-Hagin D, Gyansa-Luterrodt M, Samman E, Agyeman FKA, Appiah G et al. Children against antibiotics misuse and antimicrobial resistance: assessing effectiveness of storytelling and picture drawing as public engagement approaches. Wellcome Open Research. 2021;6. 10.12688/wellcomeopenres.16543.1 PMC8546736 34746442

[34] Ajzen I (1991). The theory of planned behavior. Organ Behav Hum Decis Process.

[35] Miller BJ, Carson KA, Keller S (2020). Educating patients on unnecessary antibiotics: personalizing potential harm aids patient understanding. J Am Board Family Medicine: JABFM.

[36] Ajzen I (2020). The theory of planned behavior: frequently asked questions. Hum Behav Emerg Technol.

