# Supplementary material for: Knowledge, attitudes and behaviour of Egyptians towards antibiotic use in the community: can we do better?
Source: Antimicrob Resist Infect Control. 2023 May 24;12:50. doi: 10.1186/s13756-023-01249-5 (PMC10210355; doi:10.1186/s13756-023-01249-5)

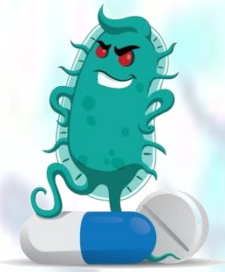

المضاد الحيوي

تفيد المضادات الحيوية في مكافحة البكتيريا وليس الفيروسات، كتلك التي تسبّب نزلات البرد والإنفلونزا. إن أخذ مضاد حيوي لا تكون بحاجة إليه لن يحسن حالتك و لن يعجل بتعافيك. عندما تبدأ بالشعور بتحسن فإن ذلك يكون عادة نتيجة قيام جهاز المناعة لديك بعمله في مكافحة العدوى التي أصابتك.

تنتج البكتيريا أو الفطريات المواد المقاومة للميكروبات بصورة طبيعية والتي يمكن أن تقتل الكائنات المجهرية الأخرى أو أن تكبح نموها. يستخدم الناس أنواعا كثيرة من المضادات الحيوية كأدوية للوقاية من العدوى التي تنقلها البكتريا المسببة للمرض والفطريات وبعض الطفيليات، ولعلاج هذه العدوى. وتستخدم أغلبية المضادات الحيوية ضد البكتيريا بصفة أساسية. نظرا لأن المضادات الحيوية هي نوع من أنواع مضادات الميكروبات، فإن المصطلحين يستخدمان عادة للتعبير عن نفس المعنى

تُعرّف البكتيريا بأنّها كائنات مجهرية وميكروبات ذات خلية واحدة، وتتميز هذه الخلية بأنّ بنيتها أبسط من بنية خلايا باقي الكائنات الحية، إذ لا توجد فيها أي نواة أو عضيات مرتبطة في الأغشية، وعوضاً عن ذلك تحتوي على مركز تحكم، يتم فيه حفظ المادة الوراثية على شكل حلقة من الحمض النووي، كما أنّ بعض أنواع البكتيريا قد تحتوي حلقة إضافية من المادة الوراثية تُسمى البلازميد ، ويحتوي البلازميد على جينات تعطي بعض أنواع البكتيريا صفات مميزة، تميزها عن باقي أنواع البكتيريا الأخرى، مثل: مقاومة نوع معين من المضادات الحيوية.

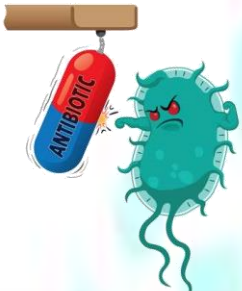

مقاومة مضادات البكتيريا

توجد البكتيريا في كل مكان في البيئة و على او داخل جسم الإنسان هناك حوالى 10000 نوع مختلف من البكتيريا داخل جسم الإنسان ، خلايا البكتيريا التي توجد داخل جسم الإنسان اكثر من خلايا الجسد الإنسانية معظم البكتيريا تُعد مفيدة او غير مُضرة لكن هناك بعض انواع البكتيريا المُضرة التي تُسبب الامراض و يتم علاجها بالمضادات الحيوية عندما تم اكتشاف المضادات الحيوية كانت شديدة الفاعلية و قد ساهمت في زيادة مُعدل حياة الإنسان ل10 اعوام اكثر. احد اهم اسباب فاعلية المضادات الحيوية هي انها مُخصصة للبكتيريا و تقضى عليها دون ان تسبب ضرر للخلايا الصحية داخل اجسادنا تُهاجم المضادات الحيوية الجسد بعدة طرق مختلفة:

1- تمنع عملية بناء جدار خلية البكتيريا.

2- تتدخل في بعض العمليات الحيوية مثل صناعة البروتين داخل الخلية.

في بعض الاحيان تتعرض البكتيريا لطفرة جينية مما يجعلها مُقاومة للمضادات الحيوية ،امثلة على طرق مقاومة البكتيريا للمضاد الحيوي (tetracycline):

- تستهدف المضادات الحيوية جزء مخصص من خلية البكتيريا، فعندما يدخل المضاد الحيوي الى الداخل، تدفعه الخلية خارجها و تمنعه من الوصول الى هدفه. كيف تنقل البكتيريا الجينات المقاومة للمضادات الحيوية:

1- البكتيريا صغيرة الحجم، سريعة الانقسام و تتشارك الجينات.

2- عندما تموت احد خلايا البكتيريا، تقوم بنشر الجينات للبيئة المحيطة بها حتى يحصل عليها الخلايا الاخرى.

3- تنتقل الجينات ايضًا خلال الفيروسات التي تُصيب البكتيريا و تدمرها ثم تقوم بنقل الجينات الى خلية اخرى من البكتيريا.

- تعتبر مقاومة البكتيريا خطر يهدد العالم بأكمله و ذلك للأسباب الاتية:

1- لم يستطع العلماء اكتشاف انواع جديدة من المضادات الحيوية منذ 1987 .

2- زراعة الاعضاء، العلاج الكيميائي للسرطان، و العمليات الجراحية المختلفة ستصبح فى غاية الصعوبة بسبب فقد المضادات الحيوية لفاعليتها.

3- الاهتمام بالأطفال سيُشكل خطر كبير بسبب الامراض التي لا يوجد لها علاج.

وفي عام 2050 سيصل عدد الوفيات الناتجة عن مقاومة المضادات الحيوية الى 100 مليون في السنة ، و هذا يتخطى الوفيات الناتجة عن السرطان.

المضادات الحيوية والبيئة

يمكن أن يؤدي تفريغ المضادات الحيوية وغيرها من المركبات المضادة للميكروبات، مثل المطهرات والمعادن الثقيلة، في البيئات الطبيعية إلى تطور البكتيريا المقاومة. و توجد هذه المركبات في المياه والتربة بنطاقات واسعة من التركيز، طبقا المصادر و للسلوك من ناحيه معدل التحلل والامتصاص في المواد الصلبة. وتحتوي مياه الصرف في البلديات على مجموعة كبيرة من الملوثات: المستحضرات الصيدلانية ومنتجات العناية الشخصية من المنازل، ونفايات المستشفيات ذات التركيزات العالية من المضادات الحيوية و المعقمات ومركبات من الأنشطة الصناعية بما في ذلك المعادن الثقيلة. تصرف بعض منشآت الإنتاج المستحضرات الصيدلانية كميات كبيرة جداً من المضادات الحيوية مباشرة إلى البيئة، مما يؤدي إلى تركيزات تصل إلى ما يستخدم لمعالجة الإصابة في البشر أو تتجاوزها.

ويُعد مستوى المقاومة المرتفع الموجود بالقرب من المنشأة دليلاً قاطع على أن الانتقاء بالنسبة لمقاومة المضادات الحيوية يحدث في البيئات الملوثة. ولكن تركيزات المضادات الحيوية في معظم النفايات السائلة والمياه السطحية وبيئات التربة يمكن أن تكون أقل بمقدار 1000 مرة من المستويات المستخدمة في العيادات أو في النفايات الصناعية السائلة غير المعالجة

إن التلوث منخفض التركيز هو المهم بصفة خاصة لأن التركيز أقل بكثير من أن يقتل البكتيريا التي تتعرض له، ولكنه يكفي انتقائها للمقاومة. وتتمثل المسألة فيالعتبة التي يكون فيها للمضادات الحيوية أثر انتقائي على المجتمعات الميكروبية. عند مستوى تركيز المضادات الحيوية المنخفض، قد يعتمد اكتساب المقاومة أكثر على انتقال الجين من كائن بكتيري آخر، وهو ما يعرف باسم الانتقال الأفقي للجينات. ولذلك فإنه من غير المحتمل أن تعطي دراسات البكتيريا التي تجري على نوع واحد على ألواح آغار فهما مجديا بشأن تطور المقاومة في المجتمعات الميكروبية المعقدة الموجودة في البيئة الطبيعية.

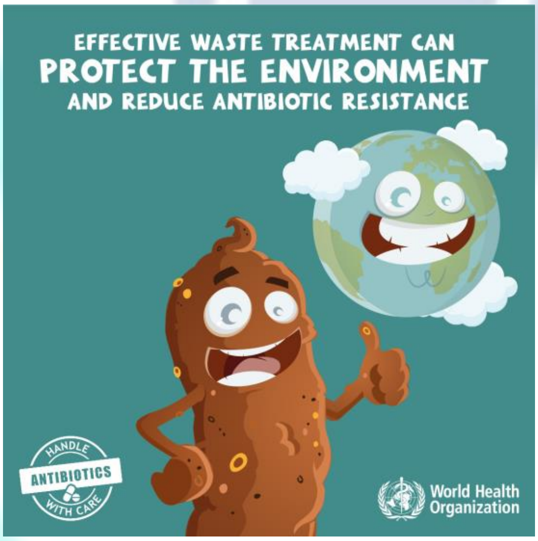

خطورة الاستخدام الخاطىء للمضادات الحيوية

وبسبب سوء استخدام المضادات الحيوية : يمكن أن تنتج مقاومة المضادات الحيوية عن تناول المضادات الحيوية بطريقة خاطئة أو عندما لا تكون هناك حاجة إليها. تتغير البكتيريا أو تتكيف مع مرور الوقت ؛ هذا ينطبق بشكل خاص على البكتيريا التي تتعرض لمضادات حيوية ولكن لا تقتل .وإن اسباب مقاومة البكتيريا هو تعرضها المستمر للمضادات الحيوية نتيجة :

1- الإستخدام الخاطئ للمضادات الحيوية.

2- استخدامها بمعدلات مرتفعة.

3- استخدام المضادات الحيوية لعلاج الإصابات الناتجة عن الفيروسات.

4- استخدامها دون استشارة الطبيب.

5- عدم اتباع الجرعة المحددة من قبل الطبيب

وطبقاً لمنظمة الصحة العالمية، فإننا قد نكون مقبلين على عصر ما بعد المضادات الحيوية حيث من الممكن أن تصبح إصابات بكتيرية بسيطة، كانت في الماضي قابلة للعلاج، قاتلة، وحيث يستحيل إجراء عمليات طبية روتينية، مثل تغيير المفاصل والعلاج الكيميائي،
والتي تعتمد على العلاج الوقائي بالمضادات الحيوية وقد قدر تقرير أونيل لعام 2014الذي كلّفت به حكومة المملكة المتحدة أن الإصابة المقاومة لمضادات الميكروبات قد تصبح السبب الرئيسي في الوفاة على مستوى العالم بحلول عام 2050

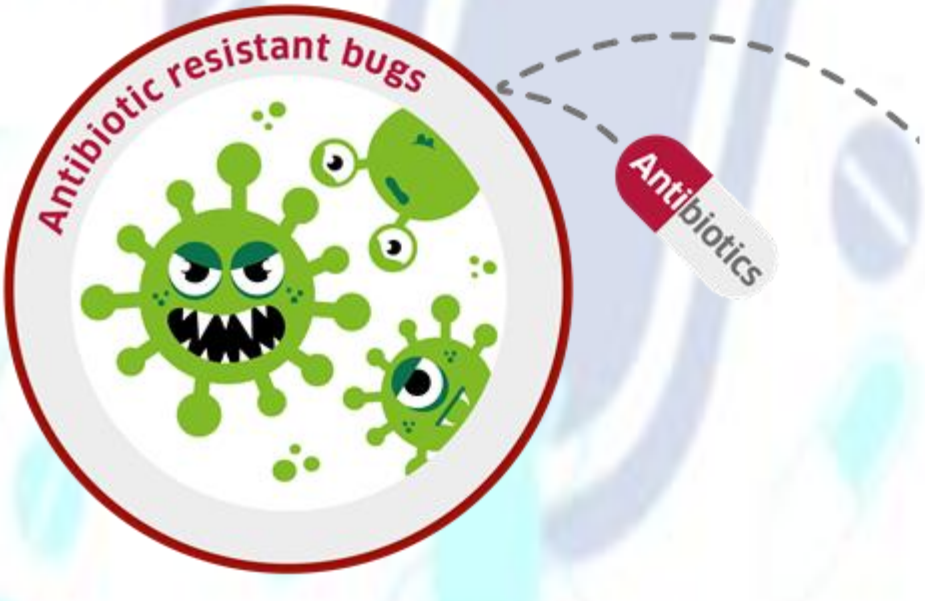

طرق الحد من الاستخدام الخاطئ

تم عمل خطة عالمية بشأن مقاومة مضادات الميكروبات:

في جمعية الصحة العالمية الثامنة والستين في مايو 2015 ، أقرت جمعية الصحة العالمية عالمية خطة عمل لمعالجة مقاومة مضادات الميكروبات ، بما في ذلك مقاومة المضادات الحيوية.
ولتحقيق هذا الهدف تم تحدد خطة عالمية تتكون من خمسة أهداف استراتيجية:

1- لتحسين الوعي وفهم مقاومة مضادات الميكروبات
2- لتعزيز المعرفة من خلال المراقبة والبحث ؛
3- للحد من حدوث العدوى.
4- لتحسين استخدام العوامل المضادة للميكروبات
5- تطوير الحالة الاقتصادية للاستثمار المستدام الذي يأخذ في الاعتبار احتياجات جميع البلدان ، وزيادة الاستثمار في الأدوية الجديدة ، وأدوات التشخيص واللقاحات وغيرها من التدخلات الأخرى

وقد استرشد تطوير هذه الخطة بمشورة البلدان وأصحاب المصلحة الرئيسيين ، بناءً على عدة مشاورات لأصحاب المصلحة المتعددين في مختلف المنتديات العالمية والإقليمية.

إرشادات أسبوع التوعية بالمضادات الحيوية لعام 2019:

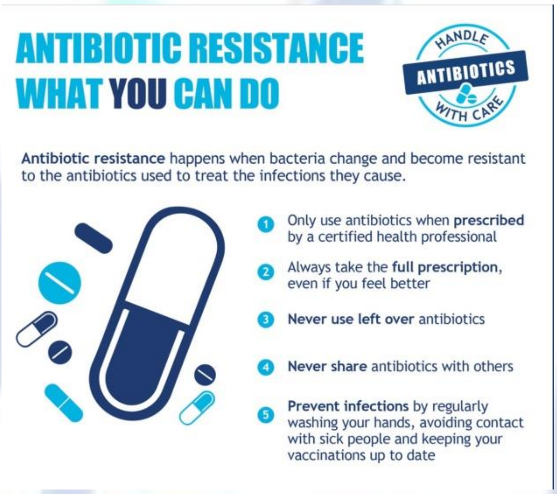

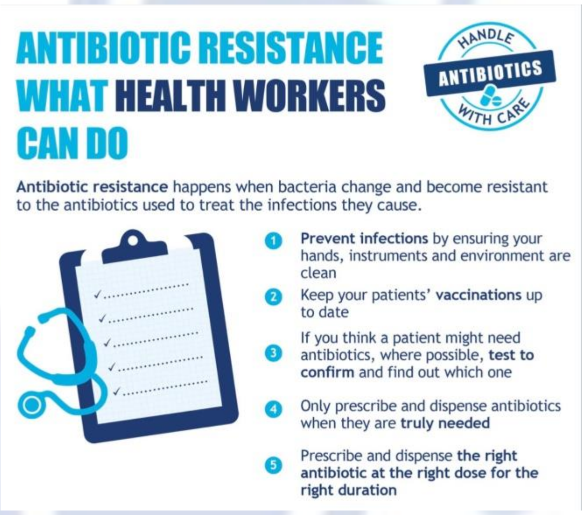

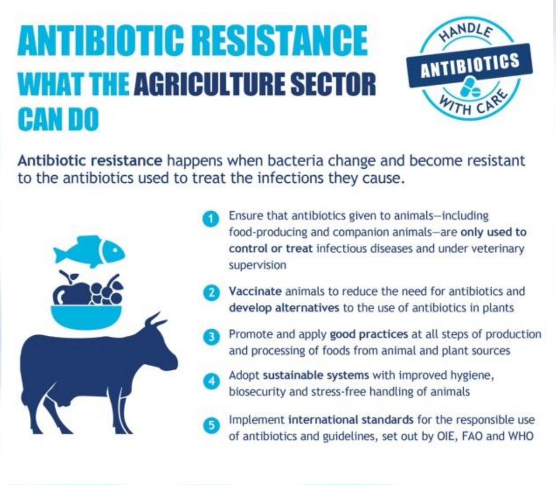

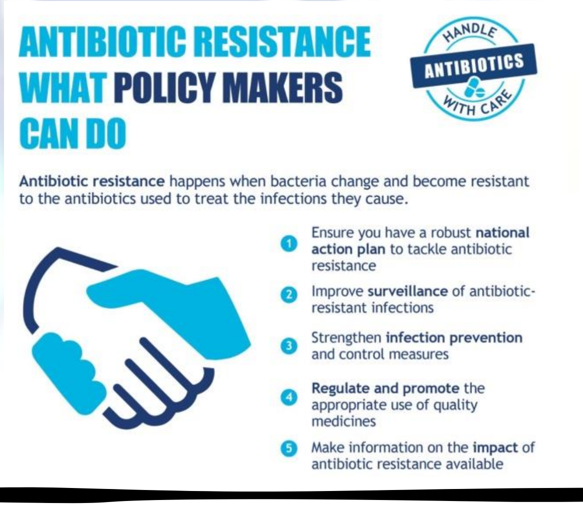

Supplement: Supplementary file 1 — Additional File 1. Examples of educational flyers used by the clinical pharmacy students in the awareness campaign. [file 13756_2023_1249_MOESM1_ESM.pdf]
