# Supplementary material for: Knowledge, attitudes and behaviour of Egyptians towards antibiotic use in the community: can we do better?
Source: Antimicrob Resist Infect Control. 2023 May 24;12:50. doi: 10.1186/s13756-023-01249-5 (PMC10210355; doi:10.1186/s13756-023-01249-5)
Supplement: Supplementary file 2 — Additional File 2. The Arabic version of the questionnaire used to assess the participants’ knowledge, attitudes and behaviour towards antibiotic use and antibiotic resistance. [file 13756_2023_1249_MOESM2_ESM.pdf]

استطلاع عن المضادات الحيوية ومقاومة المضادات الحيوية

التاريخ: ..../............./.................. (اليوم/الشهر/السنة)

نشكركم على أخذ الوقت لإكمال هذا الاستطلاع. يجب أن تعكس الإجابة على الأسئلة التي تقدمها آرائك الشخصية. لا توجد إجابات صحيحة أو خاطئة. إذا قمت بارتكاب خطأ، يرجى شطبه، ووضع علامة (√) على الإجابة المقصودة.

إقرار الموافقة:

أؤكد أنني على استعداد للمشاركة في هذا الاستطلاع، والموافقة على أن البيانات يمكن استخدامها لأغراض البحث كما هو موضح في ورقة معلومات المشاركين:

☐ نعم
☐ لا

بيانات المشارك

١. في أي فئة عمرية أنت؟

☐ ١٨-٣٠ سنة
☐ ٣١-٥٠ سنة
☐ ٥١-٧٠ سنة
☐ أكثر من ٧٠ عاماً

٢. النوع؟

☐ أنثى
☐ ذكر
☐ أفضل عدم القول

٣. ما هي أعلى درجة تعليمية حَصُلت عليها؟

☐ الدراسات العليا
☐ درجة البكالوريوس/ ليسانس
☐ درجة الثانوية
☐ درجة الإعدادية
☐ لا يوجد

الجزء الأول

١. هل تناولت المضادات الحيوية في العام الماضي؟

☐ نعم
☐ لا – انتقل **إلى السؤال ٤**
☐ لا أعرف - انتقل **إلى السؤال ٤**

٢. كم مرة تناولت المضادات الحيوية في العام الماضي؟ (١ دورة من المضادات الحيوية = ١ مرة).

☐ ١ مرة
☐ ٢ أو ٣ مرات
☐ ٤ مرات أو أكثر

٣. من نصحك بأخذ المضادات الحيوية؟ يرجى وضع علامة على كل ما ينطبق.

☐طبيب
☐صيدلي
☐ممرضة
☐ صديق
☐ فرد من العائلة
☐ قررت لنفسي
☐ الآخرين، يرجى تحديد:
....
....
....

٤. عندما تشعر بالمرض، هل حدث أن أخبرك طبيبك أنك لا تحتاج إلى مضاد حيوي لعلاج مرضك؟

☐ نعم
☐ لا

٥. هل سبق لك أن أجريت اختبار حساسية مضاد حيوي قبل البدء في استخدامه؟

☐ نعم
☐ لا

٦. ما هو نوع المضاد الحيوي التي تستخدمه عادة؟

....

الجزء ٢

١. هل تعتقد أنه عليك أخذ مضاد حيوي كلما كان لديك عدوى، مثل البرد؟

☐ نعم
☐ لا

٢. هل تعتقد أن المضادات الحيوية الأكثر تكلفة هي الأكثرعرضة لتُشعِرك بتحسن؟

☐ نعم
☐ لا

٣. هل تطلب من الطبيب أن يصف لك مضاد حيوي إذا كنت تعتقد أنك بحاجة إليه، حتى لو لم يعتقد الطبيب أنه ضروري؟

☐ نعم
☐ لا

٤. عندما تشعر بالبرد، متى تعتقد أنه يجب أن تبدأ في تناول المضادات الحيوية؟

☐ أنا لا أتناولهم
☐ عند الشعور بالأعراض الأولى للبرد
☐ بعد ظهور السعال والبلغم
☐ بعد تغير لون المخاط
☐ بعد أن يقول لي الطبيب أو الصيدلي أن آخذ واحدة
☐ لم أشعر بالبرد من قبل
☐ لا أعرف

٥. عندما تتناول المضادات الحيوية، متى تعتقد أنك يجب أن تتوقف عن العلاج بالمضادات الحيوية؟

☐ عندما أشعر بتحسن
☐ بعد أن أنهي الدورة الكاملة
☐ لم أتناول مضادات حيوية من قبل
☐ لا أعرف

٦. كم مرة سمعت عن مقاومة المضادات الحيوية؟

☐ مرات عديدة
☐ عدة مرات
☐ مرة واحدة فقط
☐ لا أتذكر سماع عن مقاومة المضادات الحيوية – انتقل **إلى السؤال ١٢**

٧. أين سمعت عن مقاومة المضادات الحيوية؟

☐ من طبيب أو ممرضة
☐ من صيدلي
☐ من الأصدقاء أو العائلة
☐ في المدرسة/الكلية/الجامعة
☐ في وسائط الإعلام (التلفزيون والإذاعة والصحف والمجلات وما إلى ذلك)
☐ أخرى، يرجى تحديد

....
....
....

٨. هل أخبرت أي شخص آخر عن مقاومة المضادات الحيوية؟

☐ نعم
☐ لا

٩. هل تغير سماع مقاومة المضادات الحيوية عن اعتقادك لعدد المرات التي يجب عليك تناول المضادات الحيوية فيها؟
☐ نعم - أعتقد أنني يجب أن تأخذ هم أكثر
☐ نعم - أعتقد أنني يجب أن تأخذ منهم أقل
☐ لا

١٠. ما مدى انتشار مقاومة المضادات الحيوية في رأيك؟

☐ وجدت في الغالب في البلدان الغنية
☐ وجدت في الغالب في البلدان الفقيرة
☐ في جميع أنحاء العالم

١١. ماذا تعتقد سيحدث إذا كنت تستخدم المضادات الحيوية دون داع؟

☐ لا شيء
☐العدوى المقاومة للمضاد الحيوية ستؤثر عليّ شخصياً
☐العدوى المقاومة المضاد الحيوية سوف تؤثر على الآخرين في المستقبل
☐ العدوى المقاومة للمضادات الحيوية سوف تؤثر علي، وعلى الآخرين في المستقبل

١٢. هل تشتري المضادات الحيوية لاستخدامها دون أن يقال لك إنك تحتاج إليها من قبل الطبيب؟

☐ نعم
☐ لا

١٣. هل تعتقد أنك يجب أن يكون لديك كامل الحرية لشراء المضادات الحيوية وقتما تريد؟

☐ نعم
☐ لا

وبهذا تنتهي الدراسة الاستقصائية. بالنيابة عن فريق البحث، شكرا جزيلا على المشاركة في هذا الاستطلاع.
