# Supplementary material for: Knowledge, attitudes and behaviour of Egyptians towards antibiotic use in the community: can we do better?
Source: Antimicrob Resist Infect Control. 2023 May 24;12:50. doi: 10.1186/s13756-023-01249-5 (PMC10210355; doi:10.1186/s13756-023-01249-5)
Supplement: Supplementary file 3 — Additional File 3. The English translation of the questionnaire used to assess the participants’ knowledge, attitudes and behaviour towards antibiotic use and antibiotic resistance. [file 13756_2023_1249_MOESM3_ESM.pdf]

Antibiotics and antibiotic resistance questionnaire

Date: …………/………../………….. (day/month/year)

Thank you for taking the time to complete this survey. The answer to the questions that you give should reflect your personal opinions. There are no right or wrong answers. If you make a mistake, please cross it out, and mark your intended answer with a tick (√).

Declaration of consent

I confirm that I am willing to take part in this survey, and consent that the data can be used for research purposes as described in the Participant Information Sheet:

☐ Yes
☐ No

Participant details

1. Which age group are you in?

   ☐ 18-30 years
   ☐ 31-50 years
   ☐ 51-70 years
   ☐ More than 70 years

2. What is your gender?

   ☐ Female
   ☐ Male
   ☐ Prefer not to say

3. What is your highest education degree?

   ☐ Post-graduate studies
   ☐ Bachelors degree
   ☐ High school degree
   ☐ Middle school degree
   ☐ None

Part 1

1. Have you taken antibiotics in the last year?

☐ Yes
☐ No – **go to question 4**
☐ I don't know – **go to question 4**

2. How many times have you taken antibiotics in the last year? (1 course of antibiotics = 1 time).

☐ 1 time
☐ 2 or 3 times
☐ 4 or more times

3. Who advised you to take the antibiotics? Please tick all that apply.

☐ Doctor
☐ Pharmacist
☐ Nurse
☐ Friend
☐ Family member
☐ I decided to myself
☐ Others, please specify: ………………………………………………………………..

4. When you have felt ill, has your doctor told you that you do not need an antibiotic to treat your illness?

☐ Yes
☐ No

5. Have you ever had an antibiotic sensitivity test done before starting an antibiotic?

☐ Yes
☐ No

6. What is the type of antibiotic you usually use?

………………………………………………………………………………………….

Part 2

1. **1.** Do you believe you have to take an antibiotic whenever you have an infection, like a cold?

   ☐ Yes
   ☐ No

2. Do you believe more expensive antibiotics are more likely to make you feel better?

☐ Yes
☐ No

3. Would you ask a doctor to prescribe an antibiotic if you believe you need one, even if the doctor did not think it was needed?

☐ Yes
☐ No

4. When you get a cold, when do you think you should start taking antibiotics?

☐ I don't take them
☐ When feeling the first symptoms of a cold
☐ After cough and sputum appear
☐ After the colour of mucous changes
☐ After a doctor or pharmacist tells me to take one
☐ I have never had a cold
☐ I don't know

5. When you have been taking antibiotics, when do you think you should stop the antibiotic treatment?

☐ When I feel better
☐ After I finish the full course
☐ I have never taken antibiotics
☐ I don't know

6. How many times have you heard about antibiotic resistance?

☐ Many times
☐ A few times
☐ Only one time
☐ I don’t remember hearing about antibiotic resistance – **go to question 12**

7. Where have you heard about antibiotic resistance?

☐ From a doctor or nurse
☐ From a pharmacist
☐ From friends or family
☐ At school/college/university
☐ In the media (television, radio, newspapers, magazines etc.)
☐ Other, please specify
…………………………………………………………………………………………..

8. Have you told anyone else about antibiotic resistance?

☐ Yes
☐ No

9. Has hearing about antibiotic resistance changed how often you think you should take antibiotics?

☐ Yes – I think I should take them more
☐ Yes – I think I should take them less
☐ No

10. How widespread do you think antibiotic resistance is?

☐ Mostly found in rich countries
☐ Mostly found in poor countries
☐ Worldwide

11. What do you think will happen if you use antibiotics unnecessarily?

☐ Nothing
☐ Antibiotic-resistant infections will personally affect me
☐ Antibiotic-resistant infections will affect others in the future
☐ Antibiotic-resistant infections will affect me, and others in the future

12. Would you buy antibiotics to use without being told you need them by a doctor?

☐ Yes
☐ No

13. Do you think you should be able to buy antibiotics whenever you want them?

☐ Yes
☐ No

That concludes the survey. On behalf of the research team, thank you very much for taking part in this survey.
